# Renal effects of dexmedetomidine during coronary artery bypass surgery: a randomized placebo-controlled study

**DOI:** 10.1186/1471-2253-11-9

**Published:** 2011-05-23

**Authors:** Kari Leino, Markku Hynynen, Jouko Jalonen, Markku Salmenperä, Harry Scheinin, Riku Aantaa

**Affiliations:** 1 Department of Anaesthesiology, Intensive Care, Emergency Care and Pain Medicine, Turku University Hospital, Turku, Finland; 2 Department of Anesthesia and Intensive Care Medicine, Helsinki University Hospital, Jorvi Hospital, Espoo, Finland; 3 Department of Anesthesiology and Intensive Care, Helsinki University Hospital, Helsinki, Finland; 4 Department of Pharmacology, University of Turku, Turku, Finland

## Abstract

**Background:**

Dexmedetomidine, an alpha_2_-adrenoceptor agonist, has been evaluated as an adjunct to anesthesia and for the delivery of sedation and perioperative hemodynamic stability. It provokes dose-dependent and centrally-mediated sympatholysis. Coronary artery bypass grafting (CABG) with extracorporeal circulation is a stressful procedure increasing sympathetic nervous system activity which could attenuate renal function due the interrelation of sympathetic nervous system, hemodynamics and renal function. We tested the hypothesis that dexmetomidine would improve kidney function in patients undergoing elective CABG during the first two postoperative days.

**Methods:**

This was a double-blind, randomized, parallel-group study. Patients with normal renal function and scheduled for elective CABG were randomized to placebo or to infusion of dexmedetomidine to achieve a pseudo steady-state plasma concentration of 0.60 ng/ml. The infusion was started after anesthesia induction and continued until 4 h after surgery. The primary endpoint was creatinine clearance. Other variables included urinary creatinine and output, fractional sodium and potassium excretion, urinary potassium, sodium and glucose, serum and urinary osmolality and plasma catecholamine concentrations. The data were analyzed with repeated-measures ANOVA or Cochran-Mantel-Haenszel test.

**Results:**

Sixty-six of 87 randomized patients were evaluable for analysis. No significant between-group differences were recorded for any indices of renal function except for a mean 74% increase in urinary output with dexmedetomidine in the first 4 h after insertion of a urinary catheter (p < 0.001). Confidence interval examination revealed that the sample size was large enough for the no-difference statement for creatinine clearance.

**Conclusions:**

Use of intravenous dexmedetomidine did not alter renal function in this cohort of relatively low-risk elective CABG patients but was associated with an increase in urinary output.

This study was carried out in 1994-1997 and was thus not registered.

## Background

Perioperative administration of alpha_2_-adrenergic agonist dexmedetomidine, has been shown to reduce anesthetic requirements, enhance hemodynamic stability and provide sedation during postoperative recovery following coronary artery bypass and other surgery [1-6]. Acute kidney injury (AKI) is a recognized complication of cardiovascular surgery and one associated with high mortality and costs-of-care [7]. The pathogenesis of AKI is multifactorial and involves hemodynamic, inflammatory and nephrotoxic components [7]. It is also known that hemodynamics, sympathetic nervous system activity and renal function are tightly interrelated. Since cardiac surgery is associated with activation of sympathetic nervous system [3], dexmedetomidine-induced sympatholysis might attenuate harmful hemodynamic events resulting in prevention of AKI. In fact, alpha_2_-adrenoceptor activation does produce some potentially renal-protective effects including inhibition of renin release, increased glomerular filtration and increased secretion of sodium and water [8,9]. Moreover, pretreatment with clonidine, the archetype of alpha_2_-adrenergic agonists, has shown beneficial renal effects after cardiac surgery [10]. In an earlier study we found that urine output tended to be greater in patients receiving dexmedetomidine than in those receiving placebo in post-coronary artery bypass grafting (CABG) patients [3].

To date, no single pharmacological regimen has conclusively proved its efficacy in preventing AKI [7] and any potential means to decrease the number of cardiac surgery patients encountering this deleterious adverse effect should be sought. We conducted a study to test the hypothesis that dexmedetomidine could protect kidney function in patients undergoing CABG with extracorporeal circulation (ECC). This study reports renal effects of dexmedetomidine compared to placebo.

## Methods

**Figure 1:**
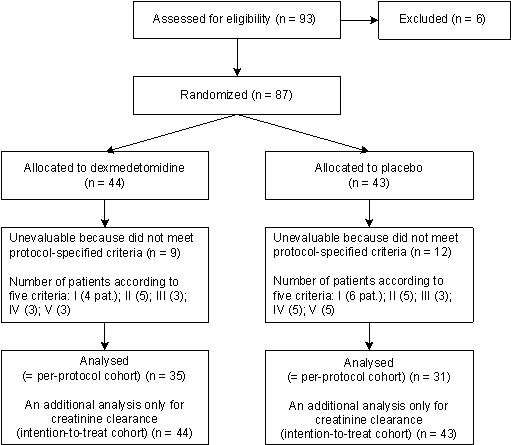
**Summary of patient disposition**.

This was a double-blind, randomized, parallel-group study designed to compare dexmedetomidine, administered as a continuous intravenous (i.v.) infusion at rates needed to achieve a pseudo steady-state plasma concentration of 0.60 ng/ml, with placebo in terms of renal effects. Altogether 93 patients were screened and 87 were randomized to receive the study treatment (Figure 1) in two study centers, Helsinki and Turku University Hospitals. Selection of the target concentration for dexmedetomidine was based on previous experience in CABG patients [3].

The study was performed according to Helsinki Declaration and standards of Good Clinical Practise. The protocol and amendments were reviewed and approved by separate Ethics Committees of Turku and Helsinki Universities and the Finnish Medicines Agency. All patients provided written informed consent. Adult (aged > 21 years) patients undergoing scheduled elective CABG surgery at the two study centers were candidates for inclusion in the study if they had normal renal function and serum creatinine (S-Crea) < 115 μmol/l, were of American Society of Anesthesiologists (ASA) physical status III or IV, and met the general participation criteria for the study. Patients were not admitted to the study if they had left main coronary artery occlusion > 50%, valvular dysfunction necessitating operation, hemodynamically significant arrhythmia, or left ventricular ejection fraction < 40%. Other exclusion conditions included: treatment with angiotensin-converting enzyme inhibitors or diuretics; use of alpha_2_-agonists to treat hypertension; untreated hypertension; cerebrovascular disease; type 1 diabetes mellitus or diabetes insipidus; morbid obesity; evidence or suspicion of abnormal liver function due to drug abuse or alcoholism; a history of severe allergy to drugs; and previous exposure to dexmedetomidine. Pregnant or breastfeeding women were ineligible, as were women of childbearing age not using reliable contraceptive methods. Randomization was performed by an independent statistician using random permuted blocks of 10 patients within each center. Both centers were provided with indistinguishable ampoules of dexmedetomidine and placebo to be allotted in numerical order to consecutive patients enrolled by study physicians. All patients, personnel and investigators including the persons responsible for data management and statistics were blinded to the treatment assignment. The code was opened only after recruitment and data collection of all study patients had been completed.

### Study outline and measurements

All patients received zopiclone 7.5 mg orally the night before surgery. Oral lorazepam 4-6 mg was administered 2 h before induction of anesthesia.

Patients were instrumented upon arrival in the operating theater, including the introduction of a pulmonary artery catheter. Ringer's acetate was infused intravenously at 10 ml · kg^-1 ^· h^-1 ^until commencement of ECC. Baseline hemodynamic and blood chemistry measurements were obtained after at least 5 min of stabilisation after catheterisation. Anesthesia was induced with 30 μg/kg fentanyl given as a 5-min i.v. infusion and co-administered with i.v. lorazepam 2 mg. Pancuronium 0.1 mg/kg was then administered i.v. and a urinary catheter introduced. All urine passed during the 48 h after catheter insertion was collected.

Study drug infusion was commenced immediately after anesthesia induction, and continued until 4 h after arrival in the intensive care unit (ICU). To reach and maintain a constant pseudo steady-state plasma concentration of 0.60 ng/ml of dexmedetomidine a five-step infusion of 4.0 μg/ml dexmedetomidine or placebo with the following decreasing infusion rates was used: 39.0 ml/h for 20 min, 24.5 ml/h for 40 min, 14.0 ml/h for 60 min, 10.5 ml/h 120 min and 7.0 ml/h from that on. The dosing was based on our previous experience with dexmedetomidine in CABG patients [3] and simulation with data available from Talke et al. [11].

CABG was performed under ECC using moderate systemic hypothermia (nasopharyngeal temperature 30°C), a standardized protocol and mannitol 150 mg/ml 100 ml added to ECC priming. ECC was initiated at a flow rate of 2.4 l min^-1 ^· m^-2 ^and reduced to 1.8 l min^-1 ^· m^-2 ^during hypothermia. Cold crystalloid potassium cardioplegic solution was used as required to maintain asystole.

Intraprocedural anaesthesia was maintained with fentanyl 0.1 μg · kg^-1 ^· min^-1 ^until rewarming (rectal temperature 35°C). Isoflurane was administered into the perfusion unit at an inspiratory concentration of 0.2%. Patients were ventilated with 40-60% oxygen in air with a target arterial CO_2 _tension of 35-45 mmHg (not corrected to the patient's temperature) and oxygen saturation (SaO_2_) > 95%. Fentanyl and isoflurane were discontinued after skin closure.

Cardiovascular parameters and clinical signs were used as indicators of anesthetic depth. Specific provisions and interventions were developed in the study protocol to maintain a sufficient level of anesthesia and acceptable hemodynamics, to prevent tachycardia, bradycardia, hypertension or hypotension lasting 1 min or more and manage low urinary output intraoperatively and postoperatively. These criteria and the respective therapeutic interventions are summarized in table 1. Deviations in other hemodynamic parameters were dealt with as a part of standard clinical practice. Post-CABG, patients were transferred to the ICU, where they were ventilated and weaned from respirator according to standard ICU protocols.

**Table 1:** Protocol-specified intraoperative and postoperative interventions for maintenance of hemodynamic and anesthetic stability, and correction of low urinary output

Tachycardia*		
Before ECC	HR > 90 beats/min	Esmolol in increments of 0.5 mg/kg i.v.
After ECC	HR > 110 beats/min	As above
In the ICU	HR > 120 beats/min	Esmolol 0.5 mg/kg i.v. If HR decreased < 120 beats/min, give metoprolol 1-5 mg i.v.
Bradycardia		
Before ECC	HR < 40 beats/min	Glycopyrrolate 0.2 mg i.v.
After ECC	HR < 70 beats/min	As above
In the ICU	HR < 60 beats/min	Pacing at 70 beats/min

Hypertension**		
Before ECC	SAP > 150 mmHg	Increase ET-IF by 0.4% and administer 50 μg i.v. bolus of glyceryl trinitrate. If response not adequate within 4 min, increase ET-IF a further 0.4% and give 5 μg/kg i.v. bolus of fentanyl. If still not adequate, increase ET-IF by 0.4% and give 50 μg i.v. bolus of glyceryl trinitrate. Taper isoflurane (to 0.2% ET-IF) when SAP and HR have reached predetermined values with no clinical signs of insufficient anaesthesia. If SAP or HR rises again, increase ET-IF by 0.4% and give 50 μg i.v. glyceryl trinitrate.
During ECC	MAP > 80 mmHg	As above
After ECC	SAP > 130 mmHg	As above
In the ICU	SAP > 150 mmHg	Start glyceryl trinitrate infusion to effect.

Hypotension		
Before ECC	SAP < 90 mmHg	Reduce ET-IF 0.4% per 4 min until ET-IF 0.2%. If not sufficient to restore SAP, administer ephedrine 2.5 mg i.v. bolus. If SAP response still not adequate repeat ephedrine bolus plus 250 ml i.v. bolus of hydroxyethylstarch (or 500 ml of Ringer's acetate). If necessary, repeat this intervention once, 4 min after first administration. If SAP response still not adequate, start epinephrine infusion at 0.03 μg · kg-1 · min-1; titrate to maximum of 0.3 μg · kg-1 · h-1. Taper epinephrine when SAP remains within protocol-specified values.
During ECC	MAP < 30 mmHg	i.v. bolus doses of phenylephrine (0.2 mg)
After ECC	SAP < 80 mmHg	As for 'Before ECC'
In the ICU	SAP < 90 mmHg	250 ml i.v. bolus of hydroxyethylstarch (or 500 ml of Ringer's acetate) over 10 min. If effective, repeat. Otherwise start epinephrine at a rate of 3 μg · kg-1 · min-1

Clinical signs of light anaesthesia	E.g. bucking, lacrimation, sweating, movement, eye opening, grimacing	As for management of hypertension

Low urinary output		
Before ECC	Urinary output < 1 ml · kg-1 · h-1 during a 30 min period, when SAP above threshold for hypotension	250 ml i.v. bolus of hydroxyethylstarch (or 500 ml of Ringer's acetate) over 10 min. If response not sufficient, repeat twice. If response not sufficient 15 min after third bolus, administer furosemide 5 mg i.v. every 30 min until effect is produced
During ECC	Urinary output < 1 ml · kg-1 · h-1 during a 30 min period, MAP > 30 mmHg	Phenylephrine 0.2 mg i.v. bolus dose, repeated up to 3 times until MAP ≥ 50 mmHg. If urinary response not sufficient despite MAP ≥ 50 mmHg for 30 min, administer furosemide 5 mg i.v. every 30 min until effect is produced
After ECC	As for 'Before ECC'	As for 'Before ECC'
In the ICU	Urinary output < 1 ml · kg-1 · h-1	250 ml i.v. bolus of hydroxyethylstarch (or 500 ml of Ringer's acetate) over 10 min. If response not sufficient, give furosemide 5 mg i.v. at 30 min intervals

*Without hypertension; **with or without tachycardia or clinical signs of inadequate anaesthesia. ECC = extracorporeal circulation; HR = heart rate;

ET-IF = end-tidal concentration of isoflurane; ICU = intensive care unit, MAP = mean arterial pressure; SAP = systolic arterial pressure.

### Creatinine clearance

The primary endpoint of the study was renal function assessed by determination of creatinine clearance at 12-24 h prior to surgery, and 0-24 h and 24-48 after urinary catheter insertion after induction of anesthesia. Creatinine clearance was defined as (U-Crea) × (urine output)/S-Crea, where U-Crea and S-Crea represent urinary and serum creatinine, respectively.

### Other variables of renal function

Creatinine clearance from 30 min urine output at 4-h intervals was quantified. Cumulative and 4-hourly urinary output was examined. Fractional sodium excretion (dU-Na) and fractional potassium excretion (dU-K) were determined at the same time as creatinine clearance. Urine samples for determination of urinary potassium (U-K), urinary sodium (U-Na), U-Crea, urinary glucose (U-Gluc) and urinary osmolality (U-Osmol) were taken after urinary catheter insertion, 1 min after aortic cross clamp removal and 24 and 48 h after urinary catheter insertion. In addition samples for determination of U-Na and U-Crea were taken at 4 h intervals until 24 h from catheter insertion.

### Plasma cathecolamines

Plasma epinephrine and norepinephrine concentrations were determined using high-performance liquid chromatography with coulometric detection [12].

### Statistical analyses

Forty patients per treatment group were needed to get a 80% power to detect a 35% difference between the treatment groups with a 5% (two-sided) type I error rate and assuming a standardized effect size (expected effect size divided by SD of the outcome variable) of 0.63. Assuming a 10% drop-out rate, the final sample size was set at 88 patients (44/group).

The analysis of renal function was based on patients meeting the following five criteria of evaluation: (i) study drug infusion commenced within 30 min after anesthesia induction and continued intraoperatively and 2-4 h postoperatively; (ii) preoperative and one other perioperative 24 h creatinine clearance value obtained; (iii) no reoperation during the study period; (iv) no development of myocardial dysfunction requiring inotropes or intra-aortic balloon pump support; and (v) extubation within 24 h post-CABG. A supplementary analysis of creatinine clearance was also carried out, based on the intention-to-treat (ITT) population of 87 patients.

Creatinine clearance was analyzed using repeated-measures ANOVA with treatment and center as grouping factors and time as a within factor. Perioperative dU-Na, total and cumulative urine volume, S-Crea, U-Crea, serum and urinary electrolytes (serum potassium [S-K], serum sodium [S-Na], urinary potassium [U-K], urinary sodium [U-Na serum and urinary osmolarity (S-Osmol, U-Osmol) and blood glucose were examined in a similar way. The Cochran-Mantel-Haenszel (CMH) test was used for urinary glucose, requirement, plasma catecholamines and requirements for postoperative cardiac pacing.

## Results

The study was carried out in years 1994-1997 and prematurely terminated due to slow recruitment rate towards the end of the study. After opening the study code it turned out that a total of 93 Caucasian patients had been screened for eligibility, of which 87 had been randomized: 44 to receive dexmedetomidine and 43 to receive placebo. Twenty-one of the randomized patients (dexmedetomidine, n = 9; placebo, n = 12) did not fulfill the evaluation criteria. Analysis of creatinine clearance was thus based on 66 evaluable patients (dexmedetomidine, n = 35; placebo, n = 31). The disposition of patients is illustrated in figure 1.

Table 2 summarizes demographic and other baseline characteristics of the evaluable patients (n = 66) in the two study groups. There were no significant differences between the groups regarding concurrent and preoperative cardiac medications, baseline laboratory data, duration of anesthesia during CABG, time to extubation or duration of study drug infusion. Mean artery pressure (MAP) was 86 ± 9 mmHg in the dexmedetomidine group and 82 ± 8 mmHg in the placebo group (p = 0.015) prior to ECC. During ECC MAP was 55 ± 7 mmHg and 58 ± 8 mmHg (p = 0.064) in dexmedetomidine and placebo groups, respectively. After ECC until admission to ICU MAP was 68 ± 6 mmHg in the dexmedetomidine group and 73 ± 9 mmHg (p = 0.019) in the placebo group. Postoperatively systolic blood pressure was measured instead of MAP up to 24 h from admission to ICU being 109 ± 10 mmHg and 111 ± 10 mmHg (p = 0.44) in dexmedetomidine and placebo groups, respectively. The total volume of intraoperative Ringer's acetate administered was 5215 ± 913 ml in the dexmedetomidine group and 4996 ± 1105 ml (p = 0.21) in the placebo group. The postoperative volumes of Ringer's acetate in the ICU up to 24 h from urinary catheter insertion were 2942 ± 1084 ml and 2958 ± 1037 ml (p = 0.41) in dexmedetomidine and placebo groups, respectively. The intraoperative hydroxyethyl starch volumes were 344 ± 309 ml and 419 ± 440 ml (p = 0.97) and postoperatively 1064 ± 385 ml and 968 ± 451 ml (p = 0.47) in dexmedetomidine and placebo groups, respectively. The average blood loss during the first 16 h after CABG was 1104 ± 376 ml with dexmedetomidine and 904 ± 307 ml with placebo (p = 0.023). Twenty-five patients in both groups needed postoperative defibrillation, equating to 71% of dexmedetomidine-treated patients and 81% of patients in the placebo group (p = 0.56 for overall comparison between groups). Eighteen dexmedetomidine patients and eight placebo patients needed postoperative pacing (p = 0.028). As this study focuses on the renal effects of dexmedetomidine and hemodynamics were strictly guided by the study protocol (Table 1), hemodynamics are not reported.

**Table 2:** Patient demographics, operation data and baseline hemodynamics

	Dexmedetomidine (n = 35)	Placebo (n = 31)
Men	31	28
Women	4	3
Age (years)	59.5 ± 8.5	62.4 ± 7.0
Weight (kg)	83.7 ± 11.9	79.7 ± 8.7
Body surface area (m^2^)	1.97 ± 0.17	1.92 ± 0.11
Systolic blood pressure (mmHg)	142 ± 21	138 ± 21
Diastolic blood pressure (mmHg)	73 ± 11	71 ± 9
Heart rate (beats/min)	61 ± 13	60 ± 7
ASA grade		
III	2	0
IV	33	31
NYHA classification		
II	12	12
III	22	17
IV	1	2
Operation time (min)	213 ± 55	190 ± 38
Perfusion time (min)	107 ± 29	96 ± 22
Number of grafts	3 ± 0.7	3 ± 0.6
Time to extubation (min)	1114 ± 248	1193 ± 283

Data are presented as number of subjects or mean ± SD. Data are based on the per-protocol cohort (n = 66). ASA = American Society of Anesthesiologists; NYHA = New York Heart Association.

### Creatinine clearance

**Figure 2:**
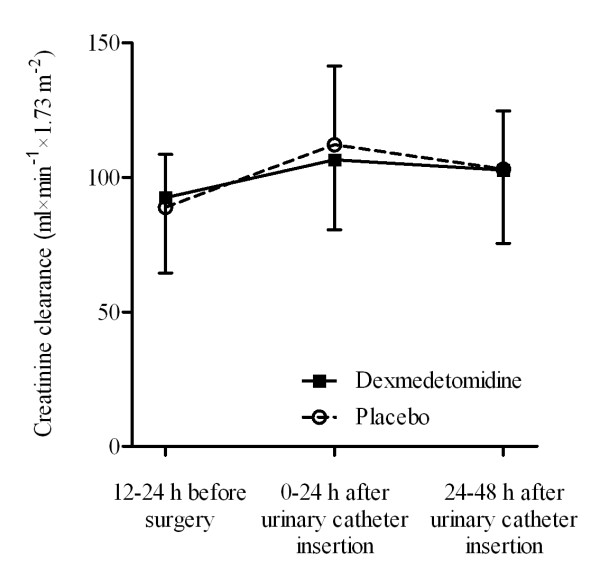
**Perioperative creatinine clearance during the period in the per-protocol cohort (primary efficacy endpoint)**. Data are expressed as mean ± SD. P < 0.001 for time-trend in both groups; no significant differences between the groups.

Perioperative creatinine clearance increased in both groups during the study period (0-24-48 h), with a significant time effect (p < 0.001) but without significant difference between the groups (p = 0.93) (Figure 2). Mean maximal increases were 15.2% and 26.2% in the dexmedetomidine and placebo groups, respectively, with no significant difference between groups. The 95% confidence intervals (CIs) for group differences were from -16.80 to 7.86 ml · min^-1 ^· 1.73 m^-2 ^in the first 24 h after catheter insertion and from -11.34 to 13.44 ml · min^-1 ^· 1.73 m^-2 ^in the second 24 h (i.e. 24-48 h after catheter insertion). Analysis based on ITT population revealed that perioperative creatinine clearance (0-24-48 h) increased in both groups similarly to evaluable patients with a significant time effect (p < 0.001). There was no significant difference between the groups (p = 0.77). Mean maximal increases were 12.4% and 20.4% in the dexmedetomidine and placebo groups, respectively, with no significant difference between groups. The 95% CIs for differences between the treatment groups were from -15.28 to 8.88 ml · min^-1 ^· 1.73 m^-2 ^in the first 24 h after catheter insertion and from -9.05 to 15.38 ml · min^-1 ^· 1.73 m^-2 ^24-48 h after catheter insertion.

### Other variables of renal function

**Figure 3:**
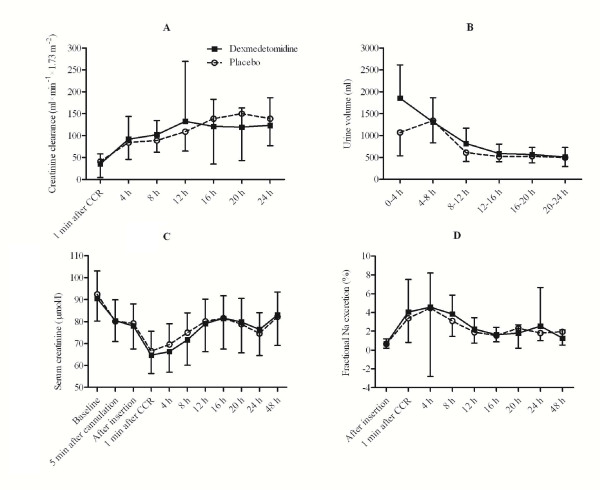
**Perioperative renal parameters**. Creatinine clearance (A) and urinary output (B) in 4-h episodes. Serum creatinine (C) and fractional sodium excretion (D) during the perioperative period. Data are expressed as mean ± SD. CCR = cross clamp removal.

There was no significant intergroup difference in creatinine clearance calculated at 4-h intervals (Figure 3A). Mean urinary output per 24 h increased from 1408 ± 623 ml before surgery to 5599 ± 1386 ml during the 24 h following catheter insertion in the dexmedetomidine group and from 1314 ± 487 ml to 4497 ± 840 ml in the placebo group (p < 0.001 between groups). Urinary volumes 24-48 h after catheter insertion were 2799 ± 757 ml and 2938 ± 1037 ml, respectively (p = 0.67). Furosemide was administered according to the conditions specified in the protocol to stimulate urine production in 22 dexmedetomidine-treated patients and 23 placebo-treated patients (p = 0.32). The median dose was 5 mg in both groups. Urinary output was also analyzed at 4-h intervals. In paired comparisons, the urinary outputs in the first (0-4 h) and third (8-12 h) fractions were significantly higher in the dexmedetomidine group when compared to placebo (p < 0.05). The mean urinary output in the first fraction was 74% greater in the dexmedetomidine group (Figure 3B; p < 0.001 vs. placebo). Fractional sodium excretion increased several-fold in both groups during and after surgery (Figure 3D).

Perioperative S-K and S-Na values were quite similar in both groups. Dexmedetomidine patients had slightly higher serum electrolyte levels towards the end of follow-up (treatment-time interactions; p = 0.0045 for S-K; p < 0.001 for S-Na) without the effect of time or treatment. Likewise, no treatment effect or effect of time in the ANOVA was noticed, but there was a treatment-time interaction for U-K (p = 0.0027). No effect of time, treatment or treatment-time interaction for U-Na was found. There was also a treatment effect on U-K (p = 0.0027 dexmedetomidine vs. placebo) at 48 h after catheter removal but not at earlier times.

Perioperative S-Crea was relatively stable, with no significant differences between the groups (Figure 3C). U-Crea first fell by > 80% from baseline in both groups thereafter gradually recovering with an overall time effect (p < 0.001). There were no overall treatment effect between the groups in S-Osmol and U-osmol (Table 3). Time-dependent changes in blood and urine concentrations of glucose were seen in both groups (p < 0.001).

**Table 3:** Serum and urine osmolality

	Dexmedetomidine		Placebo	
	**S-Osmol mosm/kg**	**U-Osmol mosm/kg**	**S-Osmol mosm/kg**	**U-Osmol mosm/kg**
	
5 min after cannulation	291 ± 4	583 ± 178	292 ± 4	625 ± 152
1 min after cross clamp removal	295 ± 4	326 ± 93	293 ± 5	353 ± 80
24 h after urinary catheter insertion	296 ± 9	618 ± 117	290 ± 7	649 ± 85
48 h after urinary catheter insertion	294 ± 7	579 ± 128	295 ± 19	506 ± 84

There were no significant differences between the groups, effect of time or treatment-time interaction in serum osmolality (S-Osmol). Urine osmolality (U-Osmol) initially decreased in both groups (p < 0.001 for time effect) without treatment effect. However, there was a significant treatment-time interaction in the ANOVA (p = 0.024) and in paired comparisons dexmedetomidine patients had significantly higher values 48 h after urinary catheter insertion (p = 0.02)

### Plasma catecholamines

**Figure 4:**
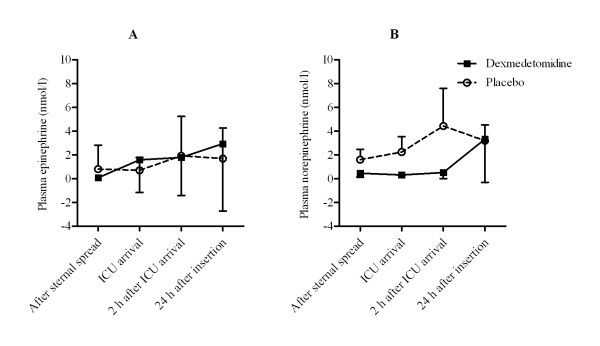
**Plasma epinephrine and norepinephrine concentrations during the perioperative period**. Data are expressed as mean ± SD.

CMH test showed a significant difference between the groups in perioperative epinephrine concentrations (p < 0.001) although the concentrations were quite similar in both treatment groups (Figure 4). Plasma norepinephrine concentrations were significantly smaller in the dexmedetomidine-treated patients (p < 0.001; Figure 4).

## Discussion

Although urinary output increased and plasma norepinephrine decreased in these relatively low-risk elective CABG patients during dexmedetomidine treatment, we were not able to demonstrate a significant dexmedetomidine-related benefit in terms of renal function as evaluated with creatinine clearance. In spite of the fact that the targeted number of evaluable patients was not reached, the confidence intervals for group differences in creatinine clearance clearly demonstrate that our sample size was adequate for a negative (no difference) conclusion. Because of smaller than anticipated variance in the primary endpoint, we can exclude a larger than 15% difference between the groups. We argue that such a small difference would hardly have clinical significance. It should also be noted that the protocol stipulated the attainment and maintenance of a dexmedetomidine plasma level of 0.6 ng/ml, which is at the lower end of the preferred therapeutic concentration [13]. Nevertheless, we did not measure the true individual plasma concentrations nor did we consider the true body weight of the patients in dose calculation. Thus, there has likely been some interindividual differences in the true plasma concentrations. Further trials may be warranted to examine the effect of higher and weight-based dexmedetomidine dosing on indices of renal function in higher risk patients.

Postoperative AKI is a relatively rare complication of CABG but has a profoundly disadvantageous impact on short-term (< 30 day) prognosis. In-hospital mortality rates of 40% and more have been reported in several series of patients, compared with rates of around 1% in patients without kidney insufficiency [14-17]. Loef et al. [17] have also reported a more than 10-fold increase in short-term mortality with postoperative kidney insufficiency and, in contrast to some other investigators [18,19], have linked postoperative AKI to reduced longer-term survival. A recent review endorses the contribution of AKI as an independent factor associated with substantial morbidity and mortality following cardiopulmonary bypass [7].

In the present study, creatinine clearance was determined by direct measurement. We are supportive of assertion of Holzmann and colleaques that indices of clearance may be preferable to serum creatinine levels as an indicator of renal function [20]. Others have advocated markers such as cystatin C as an alternative means of monitoring renal function [21]. Estimation of glomerular filtration rate might also be considered as it is part of the proposed RIFLE (risk, injury, failure, loss and end stage kidney disease) classification for determination of AKI [22]. We can state that according to urinary output and serum creatinine, there were no patients fulfilling the definition of AKI according to RIFLE in the present study.

Renal effects of alpha_2_-adrenergic agonists in clinical settings have not been studied thoroughly so far. Pretreatment with the archetypal alpha_2_-adrenergic agonists, clonidine, has shown preventive effects of renal function expressed as creatinine clearance after cardiac surgery [10]. Nevertheless, clonidine is a less selective alpha_2_-adrenergic agonist than dexmedetomidine and has somewhat different pharmacodynamic profile [1].

Attenuated response of sympathetic nervous system to CABG has been suggested as a potential benefit of alpha_2_-adrenergic agonists. Our results regarding norepinephrine support this assumption as plasma norepinephrine was reduced at various time points during the study. However, these indications of reduced sympathetic activity might have also an influence on the greater blood loss in the dexmedetomidine group compared to the placebo group, since high adrenergic output favours thrombosis. On the other hand, the relatively low number of patients and the nature of the surgery might also predispose evaluation of blood loss to type I error. More postoperative pacing was needed in dexmedetomidine group which most probably is attributable to the direct effect of dexmedetomidine on cardiac conduction times [23].

The enhancement of urinary output with dexmedetomidine during the first 24 h after catheter insertion is favourable for dexmedetomidine, though it does not necessarily indicate a preserved renal function. Nevertheless, there was an increase of creatinine clearance from baseline in both groups and the increase was 11% higher in the placebo group, though this difference did not reach statistical significance. The increase seen in both treatment groups in creatinine clearance might have been an artifact resulting from diurnal variation [24], as baseline creatinine clearance was usually determined from 12-h night time urine samples rather than from a 24-h sample. Eventual problems in spontaneous voiding might also have resulted in systematic but likely similar underestimation of baseline creatinine clearance in both groups. Increased urine output is a recognized property of dexmedetomidine and demonstrated already earlier in patients undergoing CABG by us [3] and more recently by others in patients undergoing thoracotomy [25]. The diuretic action of dexmedetomidine evident also in the present study is consistent with sympatholysis-attenuated sodium reabsorption in tubular cells via alpha_2_-adrenergic action [26]. We did not however, control or record fluid administration at the regular ward during the second postoperative day. This may have added irregularities in individual fluid management and thus urine output during that period.

The fact that we included patients at a relatively low risk of AKI may limit the interpretation of the results. In addition, we had a high drop-out rate of 21 patients due to the complexity of the study protocol. Nevertheless, all patients were included in the ITT analyses for the main end-point which were essentially the same as the per-protocol analyses.

## Conclusions

This study showed that the use of intravenous dexmedetomidine did not alter renal function in this cohort of relatively low-risk elective CABG patients although it was associated with an increase in urinary output as compared to placebo. Further trials are warranted to elucidate the effect of dexmedetomidine in high-risk patients.

## Abbreviations

Defined in the text where first used.

## Competing interests

This study was funded by Orion-Pharma (Finland). The institutions involved received an unlimited research grant from Orion-Pharma, but none of the investigators received individual fees for this study. Dr. Aantaa reports that he has been a paid consultant for both Orion Pharma and Abbott Laboratories, the co-developers of dexmedetomidine, and has received research grants from Orion Pharma. Dr. Aantaa is one of the three original authors of the dexmedetomidine patent "Use of dexmedetomidine for intensive care unit sedation". The worldwide patent rights have been transferred to Orion Pharma and Abbott Laboratories. Dr. Jalonen reports that he has received research grants from Orion Pharma for studies on dexmedetomidine and other drugs. Dr. Salmenperä reports that he has received research grants from Orion Pharma for studies on levosimendan. Dr. Scheinin reports that he has been a former employee and a paid consultant for Orion Pharma. Doctors Leino, Hynynen, Kuitunen, Valtonen, Perttilä, Heikkilä and Savunen declare that they have no competing interests.

## Author details

1) Department of Anaesthesiology, Intensive Care, Emergency Care and Pain Medicine, Turku University Hospital, Turku, Finland; 2) Department of Anesthesia and Intensive Care Medicine, Helsinki University Hospital, Jorvi Hospital, Espoo, Finland; 3) Department of Anesthesiology and Intensive Care, Helsinki University Hospital, Helsinki, Finland; 4) Department of Pharmacology, University of Turku, Turku, Finland; 5) Department of Anesthesiology, Hospital Pulssi, Turku, Finland; 6) Department of Surgery, Turku University Hospital, Turku, Finland

## Authors' contributions

KL participated in interpretation of results and writing. MH participated in the design of the study, performance of the study, interpretation of results and writing. JJ participated in the design of the study, performance of the study, interpretation of results and writing. MS participated in the design of the study, performance of the study, interpretation of results and writing. HS participated in interpretation of results and writing. RA participated in the design of the study, interpretation of results and writing. The members of the Dexmedetomidine in Cardiac Surgery Study Group (AK, MV, JP, HH, and TS) participated in performance of the study, interpretation of results and writing. All authors read and approved the manuscript and gave their intellectual contribution for it.

## Pre-publication history

The pre-publication history for this paper can be accessed here:

http://www.biomedcentral.com/1471-2253/11/9/prepub
